# Supplementation with nitrate only modestly affects lipid and glucose metabolism in genetic and dietary-induced murine models of obesity

**DOI:** 10.3164/jcbn.19-43

**Published:** 2019-11-12

**Authors:** Alexandra Fischer, Kai Lüersen, Gerhard Schultheiß, Sonia de Pascual-Teresa, Alessandro Mereu, Ignacio R. Ipharraguerre, Gerald Rimbach

**Affiliations:** 1 Institute of Human Nutrition and Food Science, Food Science, University of Kiel, Hermann-Rodewald-Strasse 6, 24118 Kiel, Germany; 2 Animal Welfare Officer, University of Kiel, Hermann-Rodewald-Strasse 12, 24118 Kiel, Germany; 3 Department of Metabolism and Nutrition, Institute of Food Science, Food Technology and Nutrition (ICTAN-CSIC), José Antonio Novais 10, 28040 Madrid, Spain; 4 Yara Iberian, C/ Infanta Mercedes 31 - 2nd floor, 28020 Madrid, Spain

**Keywords:** nitrate, mice, diet, carbohydrate and lipid metabolism, Ppar

## Abstract

To gain a better understanding of how nitrate may affect carbohydrate and lipid metabolism, female wild-type mice were fed a high-fat, high-fructose diet supplemented with either 0, 400, or 800 mg nitrate/kg diet for 28 days. Additionally, obese female *db/db* mice were fed a 5% fat diet supplemented with the same levels and source of nitrate. Nitrate decreased the sodium-dependent uptake of glucose by ileal mucosa in wild-type mice. Moreover, nitrate significantly decreased triglyceride content and mRNA expression levels of *Pparγ* in liver and *Glut4* in skeletal muscle. Oral glucose tolerance as well as plasma cholesterol, triglyceride, insulin, leptin, glucose and the activity of ALT did not significantly differ between experimental groups but was higher in *db/db* mice than in wild-type mice. Nitrate changed liver fatty acid composition and mRNA levels of *Fads* only slightly. Further hepatic genes encoding proteins involved in lipid and carbohydrate metabolism were not significantly different between the three groups. Biomarkers of inflammation and autophagy in the liver were not affected by the different dietary treatments. Overall, the present data suggest that short-term dietary supplementation with inorganic nitrate has only modest effects on carbohydrate and lipid metabolism in genetic and dietary-induced mouse models of obesity.

## Introduction

For many years, inorganic nitrate (NO_3_^−^) has been considered a hazardous chemical in food and drinking water because of it forms carcinogenic *N*-nitrosamines and causes methemoglobinemia in infants.^(1–3)^ However, recent studies also indicate a protective function of nitrate against cardiovascular disease, diabetes and metabolic syndrome.^(4,5)^ Nitric oxide (NO), which is centrally involved in the regulation of energy metabolism in mammals, is believed to mediate, at least in part, the positive effects of nitrate.^(6)^ At the physiological level, NO stimulates the uptake and oxidation of glucose and fatty acids in the skeletal muscle, heart, liver, and adipose tissue, while inhibiting the synthesis of glucose, glycogen and fat in insulin-sensitive tissues and enhancing lipolysis in white adipocytes.^(6–8)^ For these reasons, it has been hypothesized that inorganic nitrate and nitrite may serve as sources of endogenous NO with therapeutic potential against type 2 diabetes.^(4,9,10)^ Further regulatory actions of NO include stimulation of angiogenesis, blood flow, insulin sensitivity, and mitochondrial biogenesis.^(6)^ In addition, NO participates in the regulation of several physiological and metabolic functions that determine the productivity and health of farm animals, including reproduction (in females), energy metabolism, inflammation, and innate immunity.^(11)^ Moreover, animal studies have indicated that NO synthesis is upregulated in the myometrium and placenta during pregnancy.^(12,13)^

In animal cells and tissues, NO is produced from arginine through a reaction catalysed by NO synthase (Nos).^(14)^ In addition, NO is also produced from nitrate that is first reduced to nitrite and then to NO through the nitrate-nitrite-NO pathway. This pathway is carried out mainly by bacteria or a variety of enzymes and proteins, acting as nitrite reductases, including flavoproteins and Cyp450, myoglobin, xanthine oxidase and mitochondrial respiratory chain enzymes.^(15,16)^ Dietary arginine supplementation has been shown to enhance the expression of key genes for mitochondrial substrate oxidation (AMPK, *nNos*, *Pgc1α*) in diabetic rats.^(17)^ Supplementation of conventional diets with arginine for growing-finishing pigs reduced body-fat accretion, enhanced muscle gain, and improved the metabolic profile.^(18,19)^ Mice lacking the *eNos* gene developed symptoms of metabolic syndrome that were partly counteracted by feeding nitrate for 7 weeks,^(20)^ demonstrating that nitrate could partially restore the NO-deficiency in this loss-of-function mouse model. Long-term dietary nitrate/nitrite deficiency also led to the development of metabolic syndrome in mice, including endothelial dysfunction and cardiovascular death.^(21)^ Furthermore, the generation of NO by inducible *Nos* (*iNos*) in response to proinflammatory cytokines and endotoxins plays a critical role in protecting against pathogens and uncontrolled inflammation, e.g., via activation of macrophages or the modulation of the epithelial barrier function.^(22–24)^ The expression of *iNos* is increased in the skeletal muscle and adipose tissue of both genetic and diet-induced models of obesity,^(5)^ whereas inorganic nitrite has been shown to reduce mRNA levels of *iNos* as well as superoxide anion free radical production in activated macrophages.^(25)^ Increase of iNOS expression in mice paralleled impaired insulin receptor 1 and 2 expression thereby affecting insulin signal transduction.^(26)^ Contrary, a constant low production of NO is crucial for insulin secretion and β-cell function.^(27)^

Most available evidence on the regulatory roles of the nitrate-nitrite-NO pathway derives from studies dealing with food and feed naturally rich in inorganic nitrate.^(28,29)^ In the present study, we examined the effects of using Ca(NO_3_)_2_ as a source of supplemental inorganic nitrate in a genetic (*db/db*) and high-fat/high-fructose diet-induced mouse model of murine obesity. We focused on the metabolism of lipids and glucose to gain deeper insight into the potential metabolic and physiological benefits of nitrate and the underlying mechanism in the context of obesity and diabetes.

## Materials and Methods

### Experimental animals and diets

Six-week-old female C57BL/6JRj wild-type (WT) mice as well as BKS(D)-Lepr^db^/JOrlRj (*db/db*) mice were purchased from Janvier Labs (Le Genest-Saint-Isle; France). Mice had free access to tap water (nitrate/nitrite content: <1.5 mg/L) and the experimental diets throughout the experiment. Animals were housed in groups of 4 animals in Makrolon cages with wood-wool bedding within a regulated room (temperature, 22 ± 2°C; relative humidity, 50–60%; 12 h light/dark cycle).

The animal experiment was conducted in accordance with the German regulations on animal care and with permission from the responsible authority (V 241-46657/2017).

Mice were divided into six groups of 8 mice each with equal mean body weights (WT: 17.8 ± 0.96 g; *db/db*: 28.8 ± 2.02 g). WT mice were fed a purified semisynthetic, energy-dense high-fat and high-fructose diet (HFD, Ssniff S0065-E220) based on casein, corn starch and pork lard, and *db/db* mice were fed a low-fat control diet (C, Ssniff E15051, modified) based on casein, corn starch and soybean oil (Table 1). Both diets contained 0.69% arginine. After 1–2 weeks of adaptation, mice were allocated to the corresponding experimental diets, supplemented with either 0, 400 or 800 mg of inorganic nitrate/kg of diet. The source of nitrate was composed of 76% Ca(NO_3_)_2_, 7% KNO_3_, 0.8% NH_4_NO_3_, and 16.2% H_2_O (NitCal K, Yara, Norge AS, Norway). The supplementation rates were chosen to achieve a daily ingestion of 0, 60, and 120 mg of supplemental nitrate/kg of body weight, which were in line with the levels of nitrate shown elsewhere to induce our targeted metabolic phenotypes.^(28)^ Experimental diets were analysed in terms of their calcium and potassium concentration by inductively coupled plasma atomic emission spectrometry (ICP-AES) after pressure digestion (Agrolab LUFA GmbH, Kiel, Germany) according to the European standard method (DIN EN 15621:2017-10). There were no differences in calcium (0.873 ± 0.02%) and potassium (0.942 ± 0.02%) concentration between the experimental diets.

The food intake of mice was recorded daily, and body weight gain was recorded weekly. We lost two *db/db* mice; one in the 0 mg nitrate/kg diet group (db I) and one in the 400 mg nitrate/kg diet group (db II). At the end of the trial, mice were fasted for 4 h prior to anaesthesia, and blood was collected immediately from the heart. The blood concentration of glucose was measured by a glucometer (Abbott Freestyle Lite, Wiesbaden, Germany). Plasma and serum were obtained by centrifugation (3,000 × *g*, 10 min, 4°C) and stored at −80°C until analysis. Tissue samples were weighed, snap frozen and stored either at −80°C or in RNAlater (Qiagen, Hilden, Germany) at −20°C.

### Oral glucose tolerance test, body composition

After 4 weeks on the experimental diets, mice from each experimental group were fasted for 5–6 h prior to the oral glucose tolerance test (oGTT). For the oGTT, 2 g glucose/kg body weight was administered orally by gavage, and glucose levels were measured in blood taken from the tail tip (glucometer, Abbott Freestyle Lite) before and 15, 30, 60 and 120 min after glucose administration.

The body composition in WT mice was measured at the end of the feeding trial using a MiniSpec (Bruker, BioSpin MRI GmbH, Ettlingen, Germany). Fat mass, lean mass and free water were estimated in live animals using X-rays (energy settings: 45 kVp and 177 µA, voxel size: 76 µm, integration time: 300 ms, 250 projections per 180°).

### Ussing chamber

The distal ileum from WT mice was collected in ice cold KBR solution, and glucose uptake was subsequently measured in Ussing chambers (EasyMount chamber system with P2300 chambers and P2304 sliders; Physiologic Instruments, San Diego, CA) as described elsewhere.^(30)^ Glucose (10 mM) was added apically, and 10 mM mannitol was given basolaterally. The transepithelial potential difference was continuously monitored under open-circuit conditions using a DVC 1000 amplifier (WPI) and recorded through Ag-AgCl electrodes and KBR agarose bridges. The short-circuit current (ISC; µA/cm) was measured via an automatic VCC MC8 MultiChannel Voltage-Current Clamp (Physiologic Instruments) and recorded using Acquire & Analyze Data II acquisition software (Physiological Instruments).

### Histopathology

Liver tissue from WT mice (4 mice/group) was fixed in 4% and 1% formaldehyde solution. Samples were then dehydrated in a graded series of ethanol, infiltrated with methylbenzoate, and embedded in paraffin. Frontal plane serial sections of 5 µm thickness were cut and prepared for haematoxylin and eosin (H&E) staining. Pictures were taken with a Philips XL 20 (Philips, Norderstedt, Germany), and the histology was evaluated using an Axiophot with AxioCam HR (Carl Zeiss MicroImaging GmbH, Goettingen, Germany).

### Blood and liver biochemical analysis

The concentration of nitrate/nitrite (NO_2_^−^/NO_3_^−^) in blood was determined using a QuantiChrom Nitric Oxide Assay Kit (BioAssay Systems, Hayward, CA) according to the manufacturer’s instructions. Prior to the measurement, samples were deproteinated by NaOH and ZnSO_4_. The total NO_2_^−^/NO_3_^−^ content was measured by the improved Griess method, including the reduction of nitrate to nitrite by VCl_3_. Insulin (Ultra-sensitive mouse insulin Elisa Kit, Crystal Chem, Illinois) and leptin (Quantikine ELISA, R+D Systems, Abingdon, UK) in plasma were measured by ELISA using commercial kits. The alanine aminotransferase (ALT) enzyme activity in serum was evaluated fluorometrically at 37°C (Sigma-Aldrich, St. Louis). Total cholesterol and triglycerides (TG) in plasma were assayed using commercial kits (Fluitest Chol, Fluitest TG, Analyticon, Lichtenfels, Germany). For hepatic TG, lipids were extracted by homogenizing in 5% Triton-X100 and slowly heating to 80°C in a water bath for 5 min.^(31)^ The samples were cooled down and heated again, and TG in supernatants was measured. Thiobarbituric-reactive substances (TBA-RS) were analysed fluorometrically. In brief, protein in liver homogenates was precipitated by adding 5% trichloroacetic acid. After the addition of 0.5% SDS-BHT and 1% TBA, the samples were incubated for 20 min at 100°C. The formed complex was extracted in butanol and measured at 520/560 nm.

### Gene expression by quantitative real-time polymerase chain reaction

RNA was isolated from tissues using peqGOLD TriFast (PEQLAB Biotechnologie GmbH, Erlangen, Germany) following the manufacturer’s instructions. Concentrations and quality of RNA were determined and controlled with a Nano Drop 2000 (Thermo Fisher Scientific GmbH, Life Technologies, Darmstadt, Germany). Gene expression was analysed via quantitative real-time polymerase chain reaction (qRT-PCR) with a SensiFAST SYBR No-ROX One-Step Kit (Bioline GmbH, Luckenwalde, Germany) with SybrGreen detection using a Rotorgene 6000 cycler (Corbett Life Science, Sydney, Australia). Relative mRNA quantification was calculated using a standard curve. Target gene expression was normalized to the expression of the housekeeping gene Rn18S. Primers for qRT-PCR are given in Supplemental Table 1*.

### Determination of protein expression levels by Western blotting

Expression levels of AMPK and LC3 protein were measured via Western blotting in the cytoplasm fraction from fresh liver tissues as previously described.^(32)^ Protein concentrations were determined with a Pierce BCA assay (Thermo Fisher Scientific GmbH). Briefly, 30 µg of protein per sample was heated with loading buffer, denatured at 95°C for 5 min and separated by SDS-PAGE (BioRad, Munich, Germany). The fluorescence of the proteins was activated by UV exposure for 5 min before transferring the proteins onto a PVDF membrane (BioRad). Samples were blocked with skim milk dissolved in Tris-buffered saline plus 0.05% (v/v) Tween 20. Proteins (LC3, AMPK and pAMPK) were identified using respective primary antibodies (LC3: 1:500, NB100-2220, Novus Biologicals, Wiesbaden, Germany; AMPK: 1:100, sc-25792, Santa Cruz Biotechnology, Heidelberg, Germany; pAMPK (Thr172) 1:1,000, 2535, Cell Signaling, Frankfurt, Germany) and a secondary antibody (Santa Cruz Biotechnology). Protein bands were visualized with ECL reagents (Fisher Scientific, Schwerte, Germany) in a ChemiDoc XRS system (BioRad), and band intensities were calculated with Image Lab 5.0 Software (BioRad). Target protein expression was related to the total protein load per lane, assessed as PVDF membrane fluorescence.

### Analysis of fatty acid composition

Fatty acid composition was analysed in frozen liver samples by gas chromatography with a flame ionization detector as described previously.^(33)^ Fatty acid methyl esters (FAMEs) were synthesized by incubation with 0.5 M sodium methoxide, and the fatty acid composition was calculated as a percentage of the total identified FAMEs. Analysis of FAMEs was conducted in a 7820A Agilent gas chromatograph (Agilent Technologies Spain) equipped with an Agilent HP-23 capillary column (60 m × 250 µm × 0.25 µm, Agilent Technologies Spain) and helium (1.0 ml/min) as the carrier gas. The temperature protocol was as follows: initial temperature 100°C, ramp 8°C/min to 145°C (20 min), ramp 5°C/min to 195°C (5 min), ramp 5°C to 215°C (15 min), ramp 5°C to 230°C (5 min). Chromatograms were recorded and analysed using Agilent EZChrom Elite software (Agilent Technologies Spain). The method was validated with original standards for every fatty acid quantified. Additionally, tridecanoic acid (C13:0) was used as an internal standard, and in every case, a response factor of 1 was used.

### Statistical analysis

Data were analysed using one-way analysis of variance (ANOVA), followed by a post hoc test as indicated or in the case of heterogeneous variances, by the Games-Howell post hoc test. Repeated measures (oGTT, weight, energy intake) were analysed using a mixed-effects model (factor time and nitrate) after checking for sphericity, followed by the Bonferroni post hoc test. Correlation analysis (Spearman’s correlation coefficient) was conducted for selected variables. The area under the curve (AUC) in the oGTT was determined with GraphPad Prism (ver. 7.02). Unless stated otherwise, statistical analyses were performed with SPSS (ver. 24.0).

## Results

**Fig. 1:**
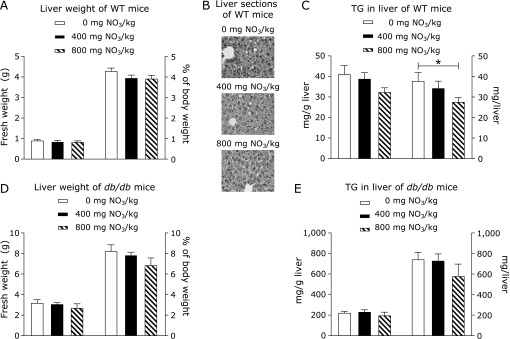
Liver fresh weight (A, D), H&E-stained sections (B, 40× magnification) and liver triglyceride (TG) content (C, E) in WT (A–C) or *db/db* mice (D, E), supplemented with either 0, 400, or 800 mg of nitrate/kg of diet. Data are means ± SEM (*n* = 8 mice/diet). Statistical analyses were performed using one-way ANOVA, followed by the LSD or Games-Howell post hoc test when variances were heterogeneous. *p<0.05.

Body weight and food intake significantly increased over time in both animal models but were not affected by nitrate supplementation (Supplemental Table 2*). Measurement of body composition in WT mice (Supplemental Table 3*) also revealed no significant differences in the proportion of body muscle, fat and water among treatment groups. Likewise, the weights of the liver (Fig. 1A and D), heart and kidney (data not shown) did not differ significantly among the groups in either animal model. H&E staining of liver samples revealed signs of hepatic steatosis in WT mice from all treatment groups (Fig. 1B), which is in line with the previously described effects of feeding high-fat/high-fructose diets.^(34)^ The hepatic content of TG in WT mice (Fig. 1C) was significantly reduced by the feeding of 800 mg nitrate/kg of diet. Even though a similar trend was observed for the same level of nitrate supplementation in *db/db* mice (Fig. 1E), differences were not significant. In addition, feeding 800 mg of supplemental nitrate increased the hepatic content of protein compared to the other treatment groups but only in *db/db* mice (Supplemental Table 3*).

The concentration of NO_3_^−^/NO_2_^−^ in blood increased in response to supplemental nitrate, reaching significance at 800 mg/kg of diet in WT mice but not in *db/db* mice (Supplemental Table 3*). The plasma levels of TG, cholesterol, insulin, and leptin as well as the activity of ALT did not differ among experimental groups but were, in all cases, many times higher in *db/db* mice than in WT mice (Supplemental Table 3*). Likewise, fasting blood glucose and hepatic TBARS, a biomarker of lipid peroxidation, were not influenced by nitrate feeding.

**Fig. 2:**
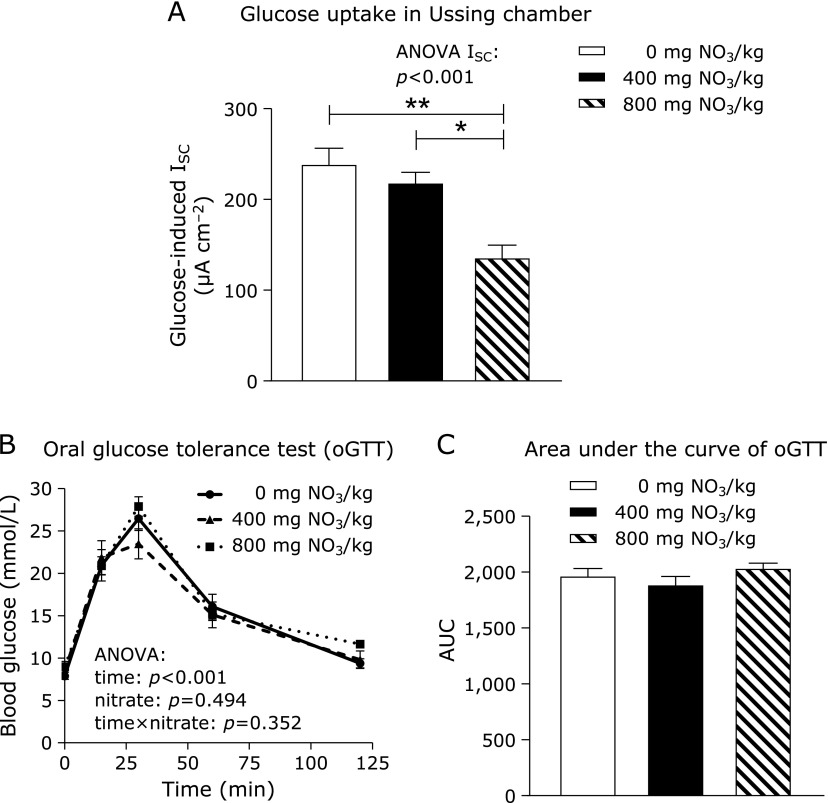
Glucose uptake *ex vivo* (A) and oral glucose tolerance test (oGTT) in WT mice (B, C) fed a high-fat/high-fructose diet supplemented with either 0, 400 or 800 mg of nitrate/kg of diet for 28 days. Data are means ± SEM (*n* = 4–8 mice/diet). Statistical analyses were performed using one-way ANOVA followed by the LSD or Games-Howell post hoc test when variances were heterogeneous. Data for oGTT were analysed using a mixed-effects method (factor time and nitrate) with repeated measures, after checking for sphericity, followed by the Bonferroni post hoc test. *p≤0.01; **p≤0.001. ISC, short-circuit current; TER, transepithelial resistance; AUC, area under the curve.

The *ex vivo* examination of glucose uptake by the ileal mucosa of WT mice revealed a significant decrease in glucose influx in the highest nitrate group compared to the other two groups (Fig. 2A). Mice from this same group, however, did not register any alteration in glycaemia over time or the area under the curve during the oGTT (Fig. 2B and C).

In *db/db* mice, blood glucose reached concentrations beyond the detection limit (30 mmol/L), rendering statistical analysis impossible. However, we observed that 15 min after glucose administration, 85.5% of mice in group I (0 mg of supplemental nitrate) were over the detection limit, whereas this percentage was 37.5 in the highest nitrate group (800 mg of supplemental nitrate). Furthermore, 60 min after glucose administration, 85.7% of mice receiving 0 mg of supplemental nitrate still had blood glucose levels over the detection limit compared to 62.5% of mice fed 800 mg of supplemental nitrate (Supplemental Table 4*).

**Fig. 3:**
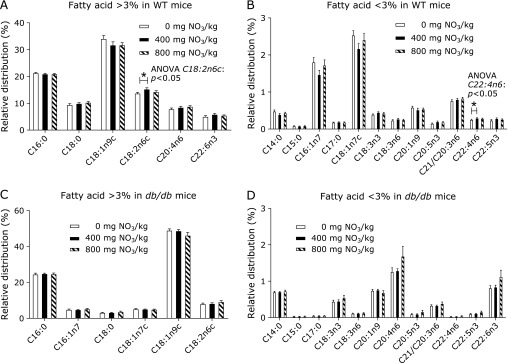
Relative fatty acid distribution in the livers of WT mice (A, B) or *db/db* mice (C, D) supplemented with either 0, 400 or 800 mg of nitrate/kg of diet. Data are means ± SEM (*n* = 7–8 mice/diet). Statistical analyses were performed using one-way ANOVA followed by the Scheffé post hoc test. *p<0.05.

In the livers of WT mice, the relative concentrations of both linoleic acid (C18:2n6c) and adrenic acid (C22:4n6) were increased by feeding 400 mg of nitrate/kg compared to those in the group receiving 0 mg of nitrate/kg (Fig. 3A). The hepatic profile of fatty acids in *db/db* mice was not affected by the content of nitrate in the diet (Fig. 3C and D).

**Fig. 4:**
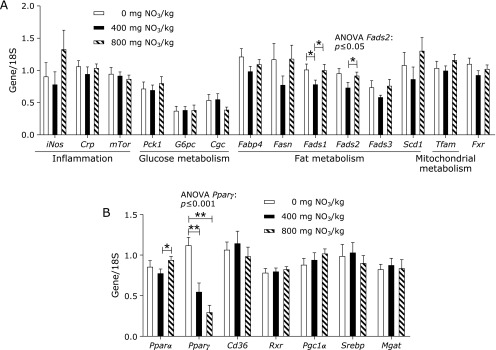
Steady-state level of hepatic mRNA (A, B) in WT mice fed a high-fat/high-fructose diet over 4 weeks, supplemented with either 0, 400 or 800 mg of nitrate/kg of diet. Data are means ± SEM (*n* = 8 mice/diet). Statistical analyses were performed using one-way ANOVA followed by the LSD or Games-Howell post hoc test when variances were heterogeneous. *p≤0.05; **p≤0.001. *iNos*, inducible nitric oxide synthase; *Crp*, c-reactive protein; *mTor*, mammalian target of rapamycin; *Pck1*, cytosolic phosphoenolpyruvate carboxykinase 1; *G6pc*, catalytic subunit of glucose-6-phosphatase; *Cgc*, glucagon (precursor of glucagon, Glp1); *Fabp4*, fatty acid binding protein4; *Fasn*, fatty acid synthase; *Fads*, fatty acid desaturase; *Scd1*, stearoyl-Coenzyme A desaturase 1; *Tfam*, mitochondrial transcription factor A; *Fxr*, farnesoid X receptor; *Pparα*, peroxisome proliferator-activated receptor α; *Pparγ*, peroxisome proliferator-activated receptor γ; Cd36, cluster of differentiation 36; Rxr, retinoid X receptor; *Pgc1α*, peroxisome proliferator-activated receptor γ coactivator 1α; *Srebp*, sterol regulatory element binding transcription factor; *Mgat*, monoacylglycerol *O*-acyltransferase; 18S, 18S ribosomal RNA.

**Fig. 5:**
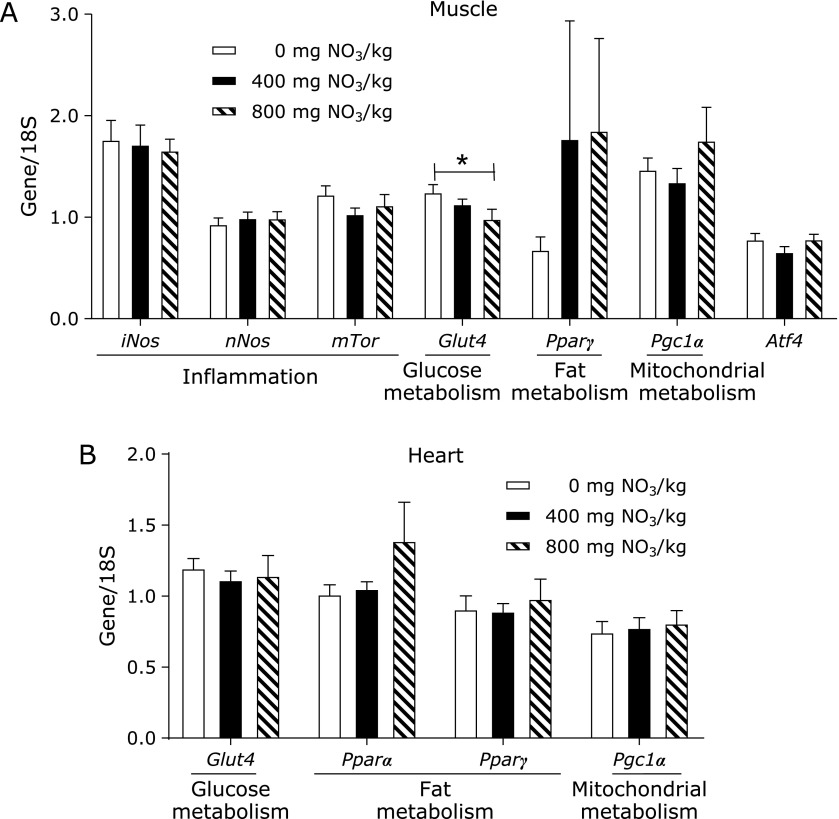
Steady-state levels of mRNA in muscle (A) or heart (B) of WT mice fed a high-fat/high-fructose diet over 4 weeks, supplemented with either 0, 400 or 800 mg of nitrate/kg of diet. Data are means ± SEM (*n* = 8 mice/diet). Statistical analyses were performed using one-way ANOVA followed by the LSD or Games-Howell post hoc test when variances were heterogeneous. *p≤0.05. *iNos*, inducible nitric oxide synthase; *nNos*, neuronal nitric oxide synthase; *mTor*, mammalian target of rapamycin; *Glut4*, glucose transporter 4; *Pparγ*, peroxisome proliferator-activated receptor γ; *Pgc1α*, peroxisome proliferator-activated receptor γ coactivator 1α;* Atf4*, *activating transcription factor 4*; *Pparα*, peroxisome proliferator-activated receptor α; 18S, 18S ribosomal RNA.

With the exception of *Fads1* and *Fads2*, whose expression was downregulated in WT mice fed 400 mg of supplemental nitrate, the mRNA steady state levels of all genes evaluated were similar among groups (Fig. 4A). We also analysed hepatic expression of *Pparα* and *Pparγ* and their related genes *Cd36*, *Rxr*, *Pgc1α*, *Srebp* and *Mgat* in the liver (Fig. 4B). The highest abundance of *Pparγ* mRNA was found in mice fed diets without supplemental nitrate and were 2-fold higher than that in mice fed 400 mg/kg of nitrate and more than 3.7-fold higher than that in mice fed 800 mg/kg of nitrate. Thus, the expression of *Pparγ* decreased with increasing nitrate content in the diet (ANOVA, *p*<0.001). Furthermore, the expression of *Pparα* was increased by nitrate supplementation at 800 mg/kg. However, we found no significant alterations in the expression of *Ppar*-related genes. It has been previously reported that skeletal muscle exhibits higher nitrate concentrations than the liver and blood and might serve as a nitrate reservoir for the direct formation of nitrite and NO.^(35)^ We therefore examined samples of muscle (Fig. 5A) and heart (Fig. 5B) from WT mice for changes in the expression of some selected genes involved in inflammation, glucose and fat metabolism and mitochondrial function. Apart from finding that the expression of *Glut4* was highest in the group receiving 0 mg of supplemental nitrate, no other differences in the mRNA levels of selected genes were evident.

**Fig. 6:**
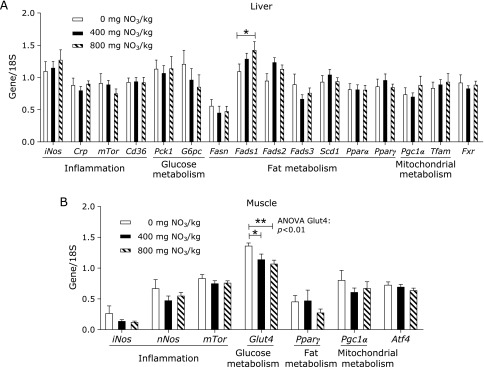
Steady-state levels of mRNA in liver (A) and muscle (B) of *db/db* mice fed a control diet over 4 weeks, supplemented with either 0, 400 or 800 mg of nitrate/kg of diet. Data are means ± SEM (*n* = 7–8 mice/diet). Statistical analyses were performed using one-way ANOVA followed by the LSD or Games-Howell post hoc test when variances were heterogeneous. *p≤0.05; **p≤0.01. *iNos*, inducible nitric oxide synthase; *Crp*, c-reactive protein; *mTor*, mammalian target of rapamycin; *Cd36*, cluster of differentiation 36; *Pck1*, cytosolic phosphoenolpyruvate carboxykinase 1; *G6pc*, catalytic subunit of glucose-6-phosphatase; *Fasn*, fatty acid synthase; *Fads*, fatty acid desaturase; *Scd1*, stearoyl-coenzyme A desaturase 1; *Pparα*, peroxisome proliferator-activated receptor α; *Pparγ*, peroxisome proliferator-activated receptor γ; *Pgc1α*, peroxisome proliferator-activated receptor γ coactivator 1α; *Tfam*, mitochondrial transcription factor A; *Fxr*, farnesoid X receptor; *nNos*, neuronal nitric oxide synthase; *Glut4*, glucose transporter 4; *Atf4*, activating transcription factor 4.

The expression of the same set of genes was measured in tissues from *db/db* mice (Fig. 6). In the liver, the expression of *Fads1* was highest in mice fed a diet supplemented with 800 mg of nitrate/kg. Mirroring findings from WT mice, the expression of *Glut4* was significantly highest in the group receiving 0 mg of supplemental nitrate. No other genes were affected by dietary nitrate in *db/db* mice.

Finally, we examined the expression of autophagy-related proteins LC3-I, LC3-II, AMPK, and pAMPK in both WT (Supplemental Fig. 1*) and *db/db* mice (Supplemental Fig. 2*). The relative intensities of bands from mice in group I (0 mg of supplemental nitrate) were set to 1. None of the examined proteins was affected by dietary nitrate supplementation either in WT or *db/db* mice.

## Discussion

Under the conditions investigated, we did not observe any differences in food intake and body weight gain in response to nitrate feeding in two models of murine obesity. A recent study by Matthews *et al.*^(36)^ similarly did not reveal any significant nitrate-related differences in body weight, or food intake or parameters of metabolic syndrome in mice fed either a high-fat diet (HFD) or a low-fat control diet (C) with or without nitrate supplementation. Additionally, no significant differences in food intake or circulating concentrations of glucose, TG, and various inflammatory markers were detected in mice after 1.5 months of feeding a low-nitrate diet compared to a nitrate-supplemented diet.^(21)^ In the same study, however, beneficial effects became evident after 3 months of nitrate feeding. Furthermore, a long-term dietary nitrite deficiency caused metabolic syndrome, endothelial dysfunction and death from cardiovascular failure in mice. It is reasonable to speculate, therefore, that the results from the present study represent rather early signs of a nitrate deficiency in the group receiving no supplemental nitrate, which might have been more pronounced under a longer feeding period of the experimental diets. This suggestion is supported by the observation that NO production declines with age due to decreased *eNos*-dependent NO synthesis.^(37,38)^ In such a context, inorganic nitrate has been shown to alleviate the senescence-related decline in liver function.^(39)^

Notably, group I, which did not receive supplemental nitrate in our study, still received nitrate, nitrite or NO (NOx) from consuming tap water (nitrate/nitrite content: <1.5 mg/L) and through dietary arginine (0.69%), the levels of which exceeded the recommendations for growing mice (<0.1% for mice, gaining 0.8 g/day, NRC).^(40)^ Furthermore, we did not observe any differences in the mRNA level of *Nos*, despite different concentrations of supplemental nitrate. In contrast, significant alterations were often reported in animals fed a complete NOx-free diet.^(21,41)^ We found an increase in the blood content of nitrate/nitrite with increasing nitrate feeding, except for the highest supplemental nitrate group in *db/db* mice. Changes in serum nitrate, nitrite and NOx levels are a controversial issue, and elevated,^(29)^ unchanged,^(20,42)^ or even decreased levels have been reported in response to dietary nitrate.^(43)^ Raat *et al.*^(44)^ evaluated the effects of supplementing drinking water with 0, 300, or 1,500 mg of nitrite/L in rats receiving deionized water and a complete NOx-free diet. The results were also compared to a control group given tap water and a control diet. Nitrite feeding led to a significant increase in plasma nitrate compared to the NOx-free rats but not compared to the control rats. The activity levels of ALT and AST was higher in the plasma of NOx-free mice. When the authors looked into differential gene expression in the liver, no differences were detected between groups using the standard analysis of differential gene expression. After applying a more statistically powerful analytic approach, they detected differences in gene expression but only between the control diet and the low-NOx diet. In addition to nitrite feeding, the authors applied ischaemia-reperfusion to the rats and observed a robust effect of nitrite on differential gene expression, confirming their hypothesis that nitrite preferentially activates gene expression during hypoxia, whereas nitrite by itself in the absence of hypoxia had little effect on gene expression. In the present study, challenging conditions were the feeding of a high-fructose/high-fat diet and the use of a genetic mouse model of obesity and type II diabetes. Other research groups also examined the effect of dietary enrichment with nitrate on HFD-induced atherosclerosis in mice compared to ApoE KO-induced atherosclerosis over 12 weeks.^(45)^ The beneficial effects of inorganic nitrate were apparent in mice fed a low-fat control diet (C) but not in those fed a HFD. The authors concluded that inorganic nitrate at the dose tested was insufficient to overcome the proinflammatory effects of a HFD. Presumably, this situation could also have been the case in our study. However, this proposition cannot be confirmed as we did not include a C-fed WT-mice group. Furthermore, the *db/db* mice exhibited a strongly defined phenotype that could not be counteracted by short-term nitrate feeding.

**Fig. 7:**
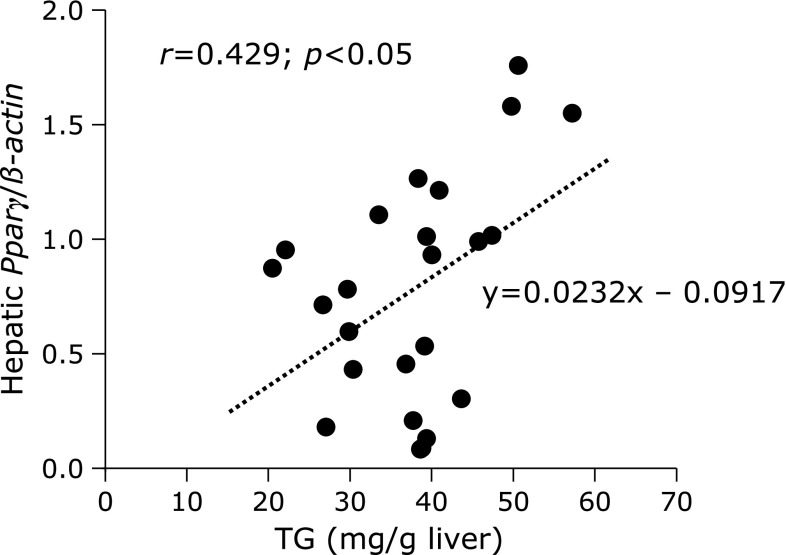
Correlation between hepatic triglyceride content and *Pparγ* mRNA level in the livers of WT mice fed a high-fat/high-fructose diet over 4 weeks, supplemented with either 0, 400, or 800 mg of nitrate/kg of diet. Spearman’s correlation analysis revealed a significant relationship of *p*<0.05. TG, triglyceride; *Pparγ*, peroxisome proliferator-activated receptor γ; *r*, Spearman’s correlation coefficient.

The liver is an important tissue for energy homeostasis and is actively involved in the synthesis, storage and redistribution of free fatty acids and glucose.^(46)^ Feeding a HFD could result in hepatic lipid accumulation, which could lead to the development of hepatic steatosis. As fructose intake is associated with non-alcoholic fatty liver disease (NAFLD) and fructose has been shown to rapidly increase hepatic TG synthesis and deposition,^(47)^ the diet of WT mice contained high fructose in addition to high fat. In the present study, the feeding of 800 mg/kg of supplemental nitrate to WT mice reduced hepatic TG and the expression of *Pparγ*, whereas it induced the expression of *Pparα*. Importantly, the mRNA levels of *Pparγ* were significantly (*r* = 0.429, *p*<0.05) correlated with hepatic TG (Fig. 7).

The mechanistic relationship between steatosis and increased *Pparγ* expression in the liver is still unclear,^(48)^ but there is a causal link between hepatic *Pparγ* expression and steatosis.^(49,50)^ Under control conditions, *Pparγ* is poorly expressed in the liver and represents only 10–30% of the level in adipose tissue.^(48,51)^ In general, *Pparγ* regulates fatty acid storage via the activation of genes that stimulate lipid uptake and adipogenesis.^(52)^ It has been shown that overexpression of *Pparγ* activates lipogenesis and increases TG in the liver.^(53)^ Conversely, a hepatocyte-specific deletion of *Pparγ* in mice was associated with a reduction in hepatic TGs and reduced expression of *Pparγ* target genes (*Cd36* and *Mgat1*) except those involved in lipogenesis (e.g., *Srebp1c*, *Fasn*, *Scd1*).^(54)^ These changes, which were most prominent when animals were fed a HFD for 14 weeks, were proposed by authors to partly account for the protection against steatosis in mice with reduced (or absent) *Pparγ* expression. In our case, therefore, it is possible that 4 weeks of feeding supplemental nitrate might have been too short to affect the expression of *Pparγ* target genes. Thus, we consider the change in expression of *Pparγ* as an early event in the beginning of steatosis. Activation of *Pparα*, which we have also seen in our study, has been shown to improve steatosis, inflammation and fibrosis in models of non-alcoholic fatty liver disease and *Pparα* agonists,^(41)^ such as fibrates, have been reported to improve fatty acid oxidation in mice.^(55)^ Furthermore, *Pparα* activation stimulates fatty acid and TG metabolism,^(49)^ thereby decreasing hepatic TG levels. Thus, simultaneous activation of *Pparα* and inhibition of *Pparγ* through nitrate supplementation may be a target for the prevention and treatment of HFD-induced steatosis.

The hepatic autophagy pathway is another mechanism involved in lipid metabolism,^(56)^ which is regulated by *Ppars* in several manners. The *Pparα*-responsive genes AMPK and *Pgc1α* are believed to activate autophagy pathways, whereas *Pparγ* seems to block them.^(57)^ Furthermore, NO may modulate AMPK activity via changes in gene expression and AMPK activation via peroxynitrite through a phosphatidylinositol 3-kinase-dependent pathway.^(17)^ On the other hand, AMPK may be able to regulate NO production through phosphorylation of *eNos* at position Ser1177.^(58)^ Even though we did not observe any significant changes in biomarkers of autophagy, there was a trend towards a higher pAMPK/AMPK ratio in animals fed 800 mg of supplemental nitrate/kg of diet (Supplemental Fig. 1 and 2*).

We found a significant reduction in glucose uptake in response to increasing levels of dietary nitrate. However, this reduction was not paralleled by improvements in oGTT, blood glucose, or insulin level, or changes in the expression of genes encoding proteins involved in glucose metabolism. Likewise, there were no significant differences in fasting plasma insulin or the intraperitoneal glucose tolerance test (iGTT) between the mice fed a nitrate-deficient diet or control diet for 1.5 months,^(21)^ whereas after 3 months, significant differences became evident. Dietary supplementation with nitrite for 10 weeks restored insulin-mediated signal transduction and improved Glut4 expression in skeletal muscles in diabetic mice but not in WT mice.^(59)^ In another study, nitrate supplementation did not significantly change iGTT, insulin, glucose or the abundance of *Glut4* mRNA and protein in control rats; however, chronic nitrate supplementation in diabetic rats significantly improved glucose homeostasis.^(43)^ In these rats, insulin secretion could not be restored to normal values by dietary nitrate. In our study, we found a downregulation of *Glut4* in muscle with increasing levels of supplemental nitrate in the diet in both murine models of obesity. Our data are in contrast to some of the published data showing increased expression of *Glut4* in response to nitrate or nitrite supplementation.^(17,59–61)^ However, Glut4 expression in isolated muscle cells from diabetic and healthy donors only increased at supratherapeutic doses of an NO donor.^(62)^ These levels of NO are higher than those achievable under physiological conditions, as in our mouse study. Another research group demonstrated that NO and nitrite stimulated *Glut4* translocation independently of insulin in *Glut4*-overexpressing cells, presumably through nitrosation of *Glut4*.^(60)^ Therefore, we cannot rule out the possibility that *Glut4* translocation was increased in our study, thereby counteracting any effect of the nitrate-induced reduction in *Glut4* expression.

In addition to the duration of nitrate feeding, structural and pharmacokinetic differences between inorganic and organic forms of nitrate might also account for the differential impact of nitrate on metabolism among studies. Inorganic nitrates are small, water-soluble ions present in the diet and are produced endogenously by oxidation of NO, whereas organic nitrates are synthetic compounds produced by nitrooxylation with more complex structures.^(63)^ It is also possible that the use of different nitrate salts among studies may have resulted in differences in the renal clearance of the anion.^(45)^ We did not collect urine, making it impossible to determine renal nitrate excretion. *In vitro* observations demonstrated that nitrate-rich beetroot could promote *Glut4* expression and stimulate myocyte metabolism through *Pgc1α* activation.^(64)^ Li *et al.*^(28)^ revealed beneficial effects of nitrate-rich spinach on insulin resistance, endothelial dysfunction and inflammation in mice fed a high-fat and high-fructose diet. In contrast to our study, nitrate was administered via gavage and fructose via drinking water, which could have led to the different outcomes between studies.

**Fig. 8:**
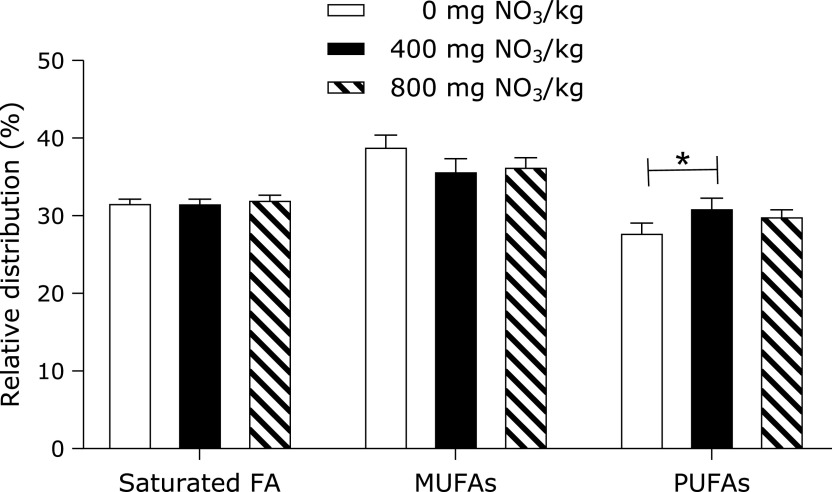
Proportion of saturated fatty acids, MUFAs and PUFAs in the livers of WT mice fed a high-fat/high-fructose diet over 4 weeks, supplemented with either 0, 400, or 800 mg of nitrate/kg of diet. Data are means ± SEM (*n* = 8 mice/diet). Statistical analyses were performed using one-way ANOVA followed by the LSD or Games-Howell post hoc test when variances were heterogonous. *p<0.05. FA, fatty acid; MUFA, monosaturated FA; PUFA, polysaturated FA.

Several studies have shown that the activities of delta-6 and delta-5-desaturases are depressed in type II diabetes.^(65,66)^ In a study by Mohan and Das,^(65)^ the intraperitoneal administration of 50 mg of l-arginine led to a significant improvement in the levels of various polysaturated fatty acids (PUFAs) in the liver, plasma and muscle of rats through enhanced activity of delta-6 and delta-5-desaturases. Similarly, we found an increase in the relative concentration of total PUFAs in the livers of WT fed 400 mg of nitrate/kg of diet (Fig. 8). The fatty acid pattern in *db/db* mice was not affected by supplemental nitrate. Altogether, nitrate feeding had only a minor influence on the hepatic profile of fatty acids in WT and *db/db* mice. It is important to consider that we used female mice, whereas most results available in the literature were derived from male rodents.^(28,43,59)^ The progression of liver steatosis and the development of insulin resistance have been shown to be sex specific, in particular due to different hormonal statuses between sexes.^(37,67)^ Likewise, male hyperlipidaemic mice developed endothelial dysfunction at an earlier age than females, which was associated with impaired NO bioavailability.^(46)^ In this latter study, the feeding of a HFD led to differential outcomes between sexes in lipid (e.g., hepatic fatty acids, *Cd36* level) and glucose metabolism (e.g., *Glut2*), *iNos* expression and nitrite/nitrate production, contributing to sex-specific differences in the development of insulin resistance.

Taken together, the results from the current study suggest that short-term dietary supplementation with inorganic nitrate has only moderate effects on carbohydrate and lipid metabolism in genetic and dietary models of murine obesity. Differences between the present study and previous literature may be related to sex, dose, type and duration of administration as well as to the total NOx content of the diet. Although we did not observe highly significant changes in lipid and glucose metabolism, supplemental nitrate tended to improve the metabolic phenotypes. In particular, the concurrent activation of hepatic *Pparα* and inhibition of *Pparγ* by supplemental nitrate suggest a potential application for the prevention and treatment of HFD-induced steatosis. Therefore, it would be interesting to investigate the impact of chronic dietary supplementation with inorganic nitrate compared to NOx-free treatment on the metabolic profile in humans and animals.

## Figures and Tables

**Table 1:** Composition of the experimental diets

	Hight-fat, high-fructose diet (WT mice)	Low-fat control diet (*db/db* mice)
Macronutrients		
Energy (MJ/kg)	22.2 MJ/kg	18.1 MJ/kg
Crude protein (%)	17.7	17.7
Arginine (%)	0.69	0.69
Crude fat (%)	23.1	5.1
Crude fibre (%)	5.5	5.5
Crude ash (%)	5.4	5.4
Starch (%)	9.6	35.6
Dextrin (%)	7.4	14.8
Sugar, total (%)	27.4	11.0
Fructose (%)	26.5	—
Supplements		
Ca(NO_3_)_2_^†^ (mg/kg diet)	0; 400 or 800	0; 400 or 800

^†^76% Ca(NO_3_)_2_, 7% KNO_3_, 0.8% NH_4_NO_3_, 16.2% H_2_O.

## Abbreviations

ALT: alanine aminotransferase
C: low-fat control diet
*Fads*: fatty acid desaturase
FAME: fatty acid methyl ester
*Glut4*: glucose transporter 4
HFD: high-fat and high-fructose diet
MUFA: monosaturated fatty acid
NO: nitric oxide
*Nos*: nitric oxide synthase
NOx: nitrate, nitrite and NO
oGTT: oral glucose tolerance test
PUFA: polysaturated fatty acid
*Ppar*: peroxisome proliferator-activated receptor
TG: triglyceride
WT: C57BL/6JRj wild-type mice
