# Supplementary material for: Supplementation with nitrate only modestly affects lipid and glucose metabolism in genetic and dietary-induced murine models of obesity
Source: J Clin Biochem Nutr. 2019 Nov 12;66(1):24–35. doi: 10.3164/jcbn.19-43 (PMC6983433; doi:10.3164/jcbn.19-43)
Supplement: Supplemental Figure 2 [file jcbn19-43sf02.pdf]

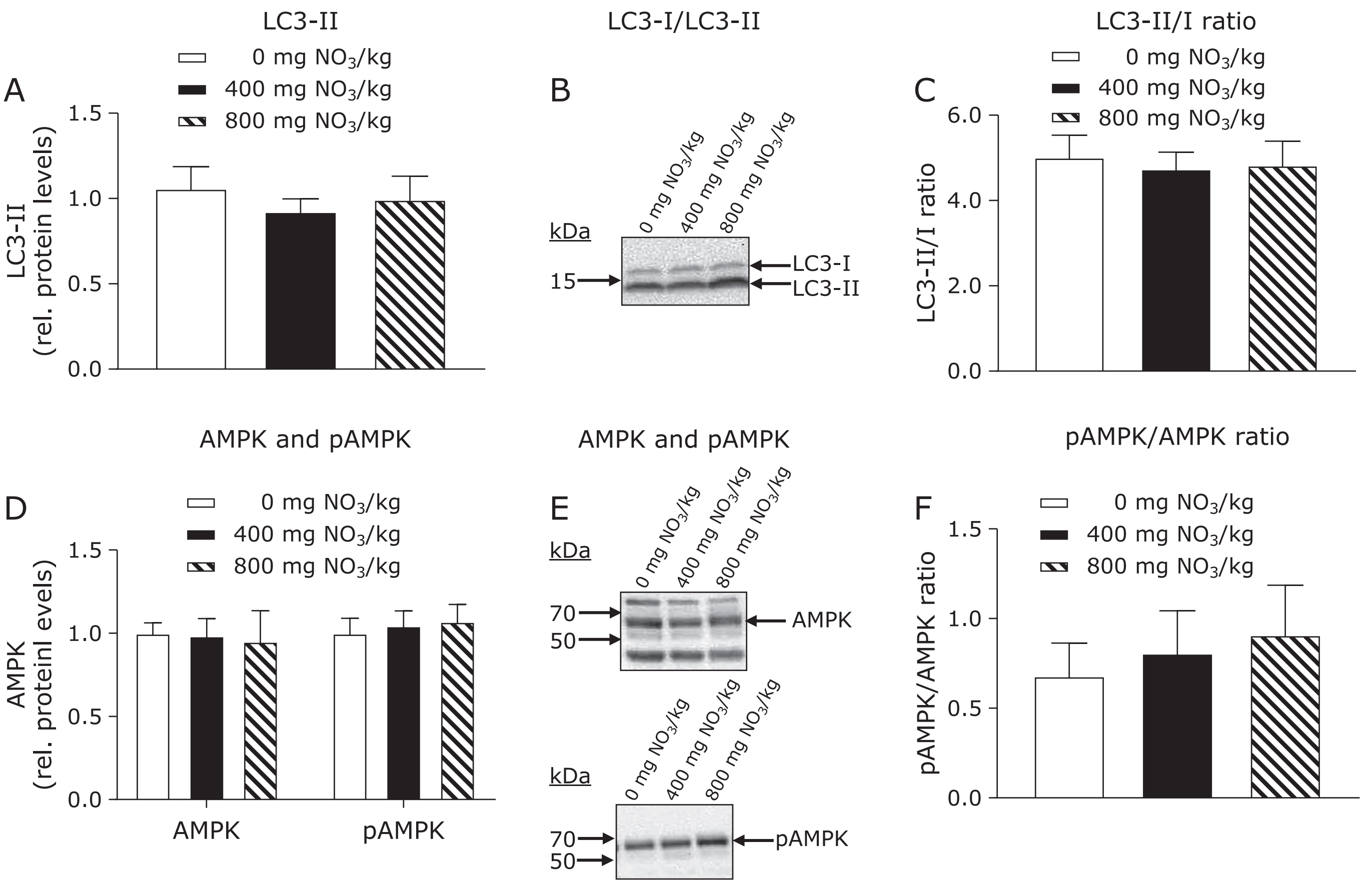

Supplemental Fig. 2. Relative protein expression levels as determined by Western blotting of LC3-II (A, B), LC3-II/I ratio (C), AMPK and pAMPK (D, E) and pAMPK/AMPK ratio (F) in liver of *db/db* mice fed a control diet over 4 weeks, supplemented with either 0, 400, or 800 mg of nitrate/kg of diet. The relative intensities of bands were quantified by densitometry, and the total protein in each lane was used as the loading control. The mean band intensity in the group with 0 nitrate supplements was set to 1. Representative Western blots are shown (B, E). Relative protein levels are presented as the means ± SEM (*n* = 8 mice/diet). Statistical analyses were performed using one-way ANOVA followed by the LSD or Games-Howell post hoc test when variances were heterogeneous. LC3, microtubule-associated proteins 1A/1B light chain 3B; AMPK, 5′adenosine monophosphate-activated protein kinase.
