# Supplementary material for: Supplementation with nitrate only modestly affects lipid and glucose metabolism in genetic and dietary-induced murine models of obesity
Source: J Clin Biochem Nutr. 2019 Nov 12;66(1):24–35. doi: 10.3164/jcbn.19-43 (PMC6983433; doi:10.3164/jcbn.19-43)
Supplement: Supplemental Table 1 [file jcbn19-43st01.pdf]

Supplemental Table 1. Nucleotide sequences of primers used in qRT-PCR analyses

Gene	Gene-ID	Description	Primer, Forward (5'-3')	Primer, Reverse (5'-3')
Atf4	11911	activating transcription factor 4	GATGATGGCTTGGCCAGT	CCAGGTCATCCATTCGAAAC
CD36	12491	cluster of differentiation 36	CAAAACGACTGCAGGTCAAC	CCAATGGTCCCAGTCTCATT
Crp	12944	c-reactive protein	AGATCCCAGCAGCATCCATA	CAGTGGCTTCTTTGACTCTGC
Fabp4	11770	fatty acid binding protein 4	AAGAAGTGGGAGTGGGCTTT	GCTCTTCACCTTCCTGTCGT
Fads1	76267	fatty acid desaturase 1	AGCACATGCCATACAACCATC	TTTCCGCTGAACCACAAAATAGA
Fads2	56473	fatty acid desaturase 2	CAGGGACTGGGTGGACTTG	ACACAAACCAGTGGCTCTCCc
Fads3	60527	fatty acid desaturase 3	AGATGAACCACATCCCCAAG	GGAAGAGGTGGTGCTCAATC
Fasn	14104	fatty acid synthase	AAGGCTGGGCTCTATGGATT	TGAGGCTGGGTTGATACCTC
Fxr	20186	farnesoid X receptor	CTCCCATTTACAGGCTACGG	GAACTTGAGGAAACGGGACA
Gcg	14526	glucagon (precursor of glucagon, Glp1)	TGAATGAAGACAAACGCCACT	CCACTGCACAAAATCTTGGGC
Glut4	20528	glucose transporter 4	CGCCTACTCAGGGCTAACAT	ATAGACTCCAAGCCCAGCAC
G6pc	14377	glucose-6-phosphatase, catalytic	GGACACCGACTACTACAGCA	TCCCAACCACAAGATGACGT
iNos	18126	inducible nitric oxide synthase	GGCAGCCTGTGAGACCTTTG	GCATTGGAAGTGAAGCGTTTC
Mgat1	68393	monoacylglycerol *O*-acyltransferase 1	CTGGTTCTGTTTCCCGTTGT	TGGGTCAAGGCCATCTTAAC
mTor	56717	mammalian target of rapamycin	CCATAAGAAAGCAGGGACCA	AGGGACACCAGCCAATGTAG
nNos	18125	neuronal nitric oxide synthase	CTGGTGAAGGAACGGGTCAG	CCGATCATTGACGGCGAGAAT
Pck1	18534	phosphoenolpyruvate carboxykinase 1, cytosolic	AGCCTTTGGTCAACAACTGG	TGCCTTCGGGGTTAGTTATG
Pgc1α	19017	peroxisome proliferator-activated receptor gamma coactivator 1α	AAGGTCCCCAGGCAGTAGAT	GCGGTATTCATCCCTCTTGA
Pparα	19013	peroxisome proliferator-activated receptor α	CCAGTACTGCCGTTTTCACA	GGCCTTGACCTTGTTCATGT
Pparγ	19016	peroxisome proliferator-activated receptor γ	AAGAGCTGACCCAATGGTTG	ACCCTTGCATCCTTCACAAG
Rn18s	19791	18S ribosomal RNA	GGTAACCCGTTGAACCCC	CAACGCAAGCTTATGACCCG
Rxr	20181	retinoid X receptor	CGCTCCATAGCTGTGAAAGA	CGTCTTGTCCATCTGCATGT
Scd1	20249	stearoyl-Coenzyme A desaturase 1	TTCTTGCGATACACTCTGGTGC	CGGGATTGAATGTTCTTGTCGT
Srebp1	20787	sterol regulatory element binding transcription factor 1	CCTAGAGCGAGCGTTGAACT	CAGAGAAACTGCAAGCAGGA
Tfam	21780	mitochondrial transcription factor A	ATTCCGAAGTGTTTTTCCAGCA	TCTGAAAGTTTTGCATCTGGGT
