# Supplementary material for: Supplementation with nitrate only modestly affects lipid and glucose metabolism in genetic and dietary-induced murine models of obesity
Source: J Clin Biochem Nutr. 2019 Nov 12;66(1):24–35. doi: 10.3164/jcbn.19-43 (PMC6983433; doi:10.3164/jcbn.19-43)
Supplement: Supplemental Table 2 [file jcbn19-43st02.pdf]

Supplemental Table 2. Body weight and food intake in WT mice (A) or *db/db* mice (B), supplemented with either 0, 400, or 800 mg of nitrate/kg of diet

A) WT mice fed a high fat/high fructose diet

Week	0	1	2	3	4	ANOVA
Body weight (g)						
WT I: HFD + 0 mg/kg nitrate	19.3 ± 1.27	19.8 ± 1.00	20.3 ± 1.49	20.8 ± 1.06	21.4 ± 0.94	time: $p<0.001$
WT II: HFD + 400 mg/kg nitrate	20.1 ± 1.59	21.1 ± 2.20	21.8 ± 2.09	22.3 ± 1.56	22.1 ± 2.01	nitrate: ns
WT III: HFD + 800 mg/kg nitrate	19.1 ± 0.55	20.1 ± 1.10	20.2 ± 0.48	21.4 ± 1.21	21.8 ± 1.39	
Food intake (g/mouse/day)†						
WT I: HFD + 0 mg/kg nitrate		1.98 ± 0.22	2.19 ± 0.62	2.22 ± 0.54	2.35 ± 0.43	time: $p<0.001$
WT II: HFD + 400 mg/kg nitrate		2.15 ± 0.40	2.20 ± 0.69	2.18 ± 0.71	1.97 ± 0.71	nitrate: ns
WT III: HFD + 800 mg/kg nitrate		2.17 ± 0.24	2.14 ± 0.66	2.36 ± 0.54	2.25 ± 0.64	

B) *db/db* mice fed a control diet

Week	0	1	2	3	4	ANOVA
Body weight (g)						
db I: C + 0 mg/kg nitrate	28.0 ± 1.51	31.5 ± 2.40	34.8 ± 2.62	37.8 ± 3.03	39.0 ± 2.90	time: $p<0.001$
db II: C + 400 mg/kg nitrate	28.3 ± 2.09	31.6 ± 2.11	35.9 ± 1.82	38.3 ± 1.78	39.3 ± 1.14	nitrate: ns
db III: C + 800 mg/kg nitrate	28.3 ± 2.47	31.5 ± 3.53	34.7 ± 4.41	37.2 ± 5.31	38.8 ± 4.83	
Food intake (g/mouse/day)†						
db I: C + 0 mg/kg nitrate		3.86 ± 0.43	4.16 ± 0.49	3.88 ± 0.56	3.62 ± 0.30	time: $p<0.001$
db II: C + 400 mg/kg nitrate		3.99 ± 0.54	4.44 ± 0.14	3.94 ± 0.35	3.67 ± 0.31	nitrate: ns
db III: C + 800 mg/kg nitrate		3.87 ± 0.41	3.97 ± 0.63	3.67 ± 0.54	3.70 ± 0.44	

HFD, high fat/high fructose diet; C, control diet; WT, wild-type mouse; db, *db/db* mouse; ns, not significant. †Due to housing in groups of 4 animals/cage, individual energy intake was calculated from the group mean. Data are means ± SD (n = 7–8 mice/diet). Significant differences were calculated using the method of mixed ANOVA (factor time and nitrate) with repeated measures after checking for sphericity, followed by the Bonferroni post hoc test.
