# Supplementary material for: Supplementation with nitrate only modestly affects lipid and glucose metabolism in genetic and dietary-induced murine models of obesity
Source: J Clin Biochem Nutr. 2019 Nov 12;66(1):24–35. doi: 10.3164/jcbn.19-43 (PMC6983433; doi:10.3164/jcbn.19-43)
Supplement: Supplemental Table 3 [file jcbn19-43st03.pdf]

Supplemental Table 3. Body composition, blood and liver parameters in WT mice (A) or *db/db* mice (B), supplemented with either 0, 400, or 800 mg of nitrate/kg of diet

A) WT mice fed a high fat/high fructose diet

	WT I (0 mg/kg nitrate)	WT II (400 mg/kg nitrate)	WT III (800 mg/kg nitrate)
Body composition			
Muscle (%)	68.5 ± 7.28	66.8 ± 7.52	67.9 ± 8.87
Fat (%)	22.6 ± 7.33	24.2 ± 7.48	22.8 ± 8.98
Water (%)	8.91 ± 0.54	9.02 ± 0.55	9.26 ± 0.41
NO_2^-/NO_3^- (mM)[†]	29.3 ± 16.7[a]	46.2 ± 3.64[a]	94.1 ± 2.4[b]
Glucose (mmol/L blood)	11.9 ± 2.39	9.3 ± 4.35	10.1 ± 3.72
Cholesterol (mg/dl plasma)	161 ± 20.4	155 ± 13.4	157 ± 19.1
TG (mg/dl plasma)	87.1 ± 14.6	81.6 ± 23.2	92.7 ± 26.1
Insulin (ng/ml serum)	0.597 ± 0.17	0.507 ± 0.14	0.617 ± 0.19
Leptin (ng/ml serum)	8.77 ± 5.85	9.32 ± 4.95	10.4 ± 7.08
ALT (mU/ml)	4.66 ± 1.60	5.01 ± 2.31	4.89 ± 3.01
Protein (mg/g liver)	124.8 ± 12.9	119.9 ± 9.47	127.9 ± 15.0
TBA-RS (nM/g liver)	4.50 ± 1.05	4.77 ± 0.86	4.33 ± 0.68

B) *db/db* mice fed a control diet

	db I (0 mg/kg nitrate)	db II (400 mg/kg nitrate)	db III (800 mg/kg nitrate)
NO_2^-/NO_3^- (mM)[†]	13.1 ± 5.04	60.5 ± 24.3	23.2 ± 1.65
Glucose (mmol/L blood)	11.9 ± 3.89	10.3 ± 7.39	11.2 ± 4.92
Cholesterol (mg/dl plasma)	298 ± 27.3	305 ± 36.5	281 ± 48.4
TG (mg/dl plasma)	182 ± 29.6	139 ± 32.6	163 ± 46.8
Insulin (ng/ml serum)	15.1 ± 8.94	8.91 ± 5.11	9.99 ± 5.44
Leptin (ng/ml serum)	179 ± 23.3	194.9 ± 13.7	198.1 ± 23.0
ALT (mU/ml)	119.4 ± 74.0	109.8 ± 30.4	77.7 ± 18.1
Protein (mg/g liver)	111.5 ± 7.54[a]	112.7 ± 8.86[a]	138.9 ± 27.3[b]
TBA-RS (nM/g liver)	2.35 ± 0.57	2.20 ± 0.20	2.73 ± 0.73

TG, triglyceride; ALT, alanine aminotransferase; TBA-RS, thiobarbiturate-reactive substances. [†]NO_2^-/NO_3^- content was measured in pooled samples. Mean group values with different superscript letters are significantly different from the other mean group values (p<0.001 for NO_2^-/NO_3^- in WT mice, ANOVA: p<0.001; p<0.05 for protein in *db/db* mice). Data are means ± SD (n = 5–8 mice/diet). Statistical analyses were performed using one-way ANOVA followed by the Scheffé or Games-Howell post hoc test when variances were heterogeneous.
