# Supplementary material for: Supplementation with nitrate only modestly affects lipid and glucose metabolism in genetic and dietary-induced murine models of obesity
Source: J Clin Biochem Nutr. 2019 Nov 12;66(1):24–35. doi: 10.3164/jcbn.19-43 (PMC6983433; doi:10.3164/jcbn.19-43)
Supplement: Supplemental Table 4 [file jcbn19-43st04.pdf]

Supplemental Table 4. Oral glucose tolerance test (oGTT) in *db/db* mice fed a control diet over 4 weeks, supplemented with either 0, 400, or 800 mg of nitrate/kg of diet

	time (min)	0	15	30	60	120
db I (0 mg/kg nitrate)	(*n*/total *n*)‡	0/7†	6/7	5/7	6/7	2/3
	(%)§	0	85.5	71.4	85.7	66.7
	Glu (mmol/L)¶	14.4	17.2	23.0	16.6	6.5
db II (400 mg/kg nitrate)	(*n*/total *n*)‡	0/8	5/8	5/8	5/7†	2/3
	(%)§	0	62.5	62.5	71.4	66.7
	Glu (mmol/L)¶	13.7	24.5	25.1	19.9	27.2
db III (800 mg/kg nitrate)	(*n*/total *n*)‡	0/8	3/8	6/8	5/8	1/4
	(%)§	0	37.5	75	62.5	25.0
	Glu (mmol/L)¶	15.1	26.3	22.8	19.9	20.1

Glu, glucose; db, *db/db* mice. †1 animal died; ‡Ratio of *n* numbers of animals with blood glucose levels above detection limit (>30 mmol/L) to total *n* numbers of animals per group. §% animals with blood glucose levels above the detection limit (>30 mmol/L) to total animals per group. ¶Mean value of blood glucose levels (mmol/L) of remaining animals.
